# Musical neglect training for chronic persistent left hemispatial neglect with right hemiplegia post-stroke: a case report

**DOI:** 10.3389/fresc.2024.1462978

**Published:** 2025-01-07

**Authors:** Yuka Kasuya-Ueba, Koji Maeda

**Affiliations:** ^1^ Department of Neuropsychiatry, Kochi Medical School, Kochi University, Kochi, Japan; ^2^ Department of Rehabilitation, Shiragikuen Hospital, Kochi, Japan

**Keywords:** case report, post-stroke, severe hemispatial neglect, chronic phase, musical neglect training, spatial attention, attention control

## Abstract

A 69-year-old right-handed man, who initially suffered a stroke 8 years ago and experienced two recurrences since then, presented with right hemiplegia and left hemispatial neglect as a post-stroke syndrome in the chronic phase. This report demonstrates the use of active musical instrument playing with Musical Neglect Training (MNT®) to improve severe left-side neglect and activities of daily living (ADLs). In addition to physical and occupational therapy, individual MNT® was incorporated into the patient's rehabilitation plan to improve his hemispatial neglect. At the initiation of the intervention, the number of uncrossed lines on the line cancellation test was 33 out of 40, and his Mini-Mental State Examination score was 17. Regarding ADLs, egocentric neglect was observed, especially during eating and wheelchair operations. Over the course of 18 months of weekly individual MNT®, a remarkable improvement was observed in the line cancellation test score (number of uncrossed lines = 4) and in ADLs. Follow-up tests showed that the effects of the intervention lasted at least 6 months. This is the first reported case demonstrating long-term effects observed at 6 months after an 18-month intervention period employing MNT® in a patient with severe chronic persistent hemispatial neglect. While rigorous studies are needed, our findings encourage further investigation of the benefits of MNT® interventions in post-stroke rehabilitation. In summary, long-term intervention involving active musical instrument playing, using auditory stimulus cues, significantly improved the severe symptoms of left hemispatial neglect in a patient with right hemiplegia, even during the chronic phase of recovery.

## 1 Introduction

Hemispatial neglect is a neuropsychological condition affecting approximately 30%–50% of stroke survivors (1, 2). It results from significant right hemisphere damage, particularly to the medial right temporal and right parietal lobes, leading to neglect in the left lateral hemispace (3). This condition is the most common symptom of higher brain dysfunction after right hemisphere injury, causing various challenges in activities of daily living (ADLs) (1, 3).

Several therapeutic approaches exist for hemispatial neglect (4), but current evidence does not definitively support any specific method (5). Active musical instrument playing and passive music listening reportedly improve symptoms of hemispatial neglect (6). Soto et al. (7) examined three patients with visual neglect following right hemisphere strokes. They assessed performance on visual tasks sensitive to neglect while patients listened to preferred music, unpreferred music, or experienced silence. Results showed that patients performed significantly better with preferred music, reducing visual neglect symptoms. Similarly, Kaufmann et al. (8) found that listening to preferred music with auditory spatial cueing significantly decreased neglect severity. These studies suggest that using preferred music during rehabilitation can enhance arousal, mood, and attention to the neglected side.

Neurologic music therapy®, grounded in the scientific principles of music perception and cognition, posits that auditory stimuli and music can positively influence sensorimotor, cognitive, and speech and language functions (9). A specific technique within neurologic music therapy® for patients with visual hemispatial neglect is Musical Neglect Training (MNT)®. MNT® uses active musical exercises on musical instruments that are “structured in time, tempo, and rhythm, and use appropriate spatial configurations of the musical instruments to focus attention on a neglected or inattentively viewed visual field” (10). MNT® design capitalizes on the brain's intrinsic ability to anticipate and complete musical patterns. This cognitive process is harnessed to promote spatial awareness and active exploration of the neglected side, effectively expanding the patient's attention field through musical engagement. Bodak et al. (11) and Kang and Thaut (12) examined the effectiveness of MNT® using tone bars, which can be arranged individually, allowing for customized placement of each note. Bodak et al. (11) conducted four weekly sessions for two patients with chronic neglect (5 and 4 years post-stroke). The Mesulam shape test revealed significant improvement for both patient 1 (pre-intervention: 27; post-intervention: 13) and patient 2 (pre-intervention: 6; post-intervention: 2). Kang and Thaut (12) implemented six twice-weekly sessions for two mild chronic neglect patients (26 months and 10 years post-stroke). Both patients demonstrated slight improvement on the line cancellation test: the pre-intervention, post-intervention, and 1-week follow-up scores were 14, 15, and 14 for patient 1 and 10, 8, and 7 for patient 2, respectively. Both studies showed that MNT®, which requires patients to play spatially appropriate musical instruments, may be a beneficial approach for chronic hemispatial neglect.

Bernardi et al. (13) investigated whether auditory feedback from a music scale could enhance spatial exploration on a musical keyboard in patients with left spatial neglect compared to a silent keyboard or one with randomly ordered pitches. They measured the number and timing of key presses, finding that patients performed better with structured sound feedback than in the other conditions. This study highlights the importance of predictable musical sequences in hemispatial neglect training, along with the positive effects of preferred music on mood and arousal. Active instrument playing may also stimulate both hemispheres, potentially enhancing interconnectivity (6, 12, 14–16).

We report on the successful use of MNT® for improving severe post-stroke left hemispatial neglect in the chronic phase. After 18 months of therapy involving active instrument playing, the patient showed significant improvements in the line cancellation test score and ADLs, which persisted for 6 months post-intervention. To our knowledge, this is the first report of long-lasting positive effects of MNT® in a patient with chronic severe hemispatial neglect. While this single case study limits the generalizability of the findings, it underscores the potential efficacy of MNT® for patients with severe chronic hemispatial neglect and may serve as a foundation for future research. This case report provides valuable insights into the application of MNT® in clinical practice and highlights the need for further investigation in this field.

## 2 Case description

**Figure 1:**
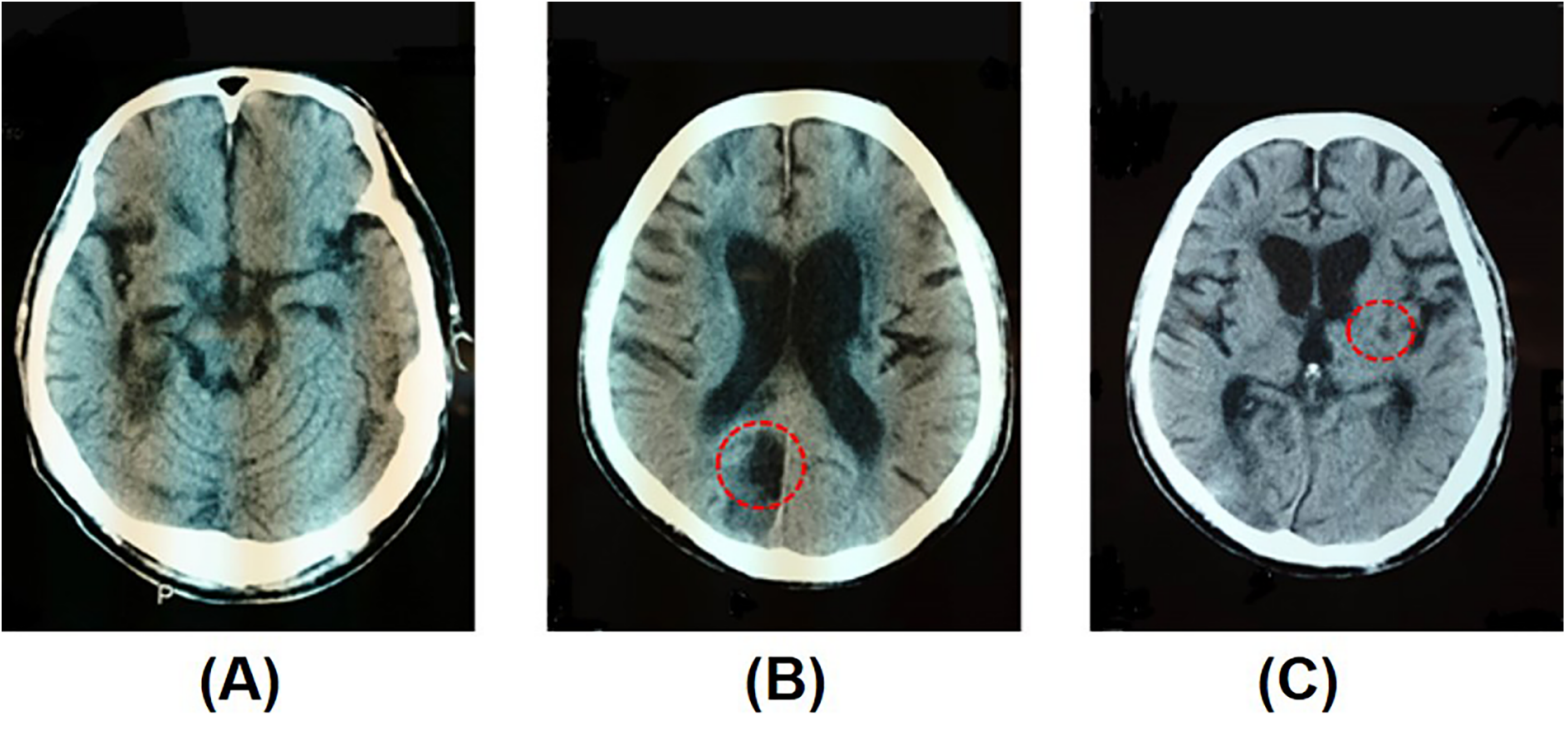
Brain computed tomography (CT) after the patient's third stroke. **(A)** No significant atrophy of the cerebrum, **(B)** infarction of the medial right occipital lobe, and **(C)** infarction of the left genu of the internal capsule (red circles).

**Figure 2:**
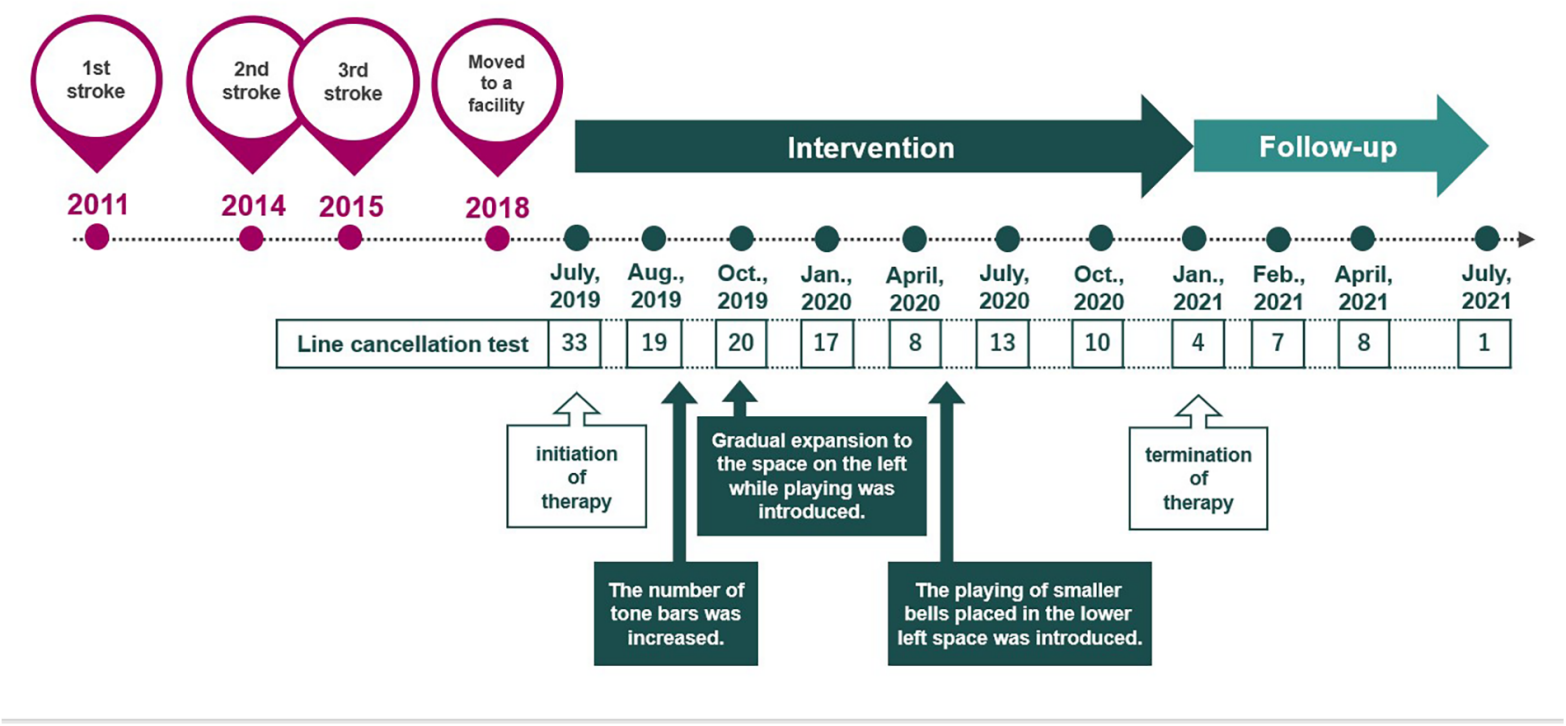
Timeline of the case, including the patient's stroke history, step-by-step approach implemented in response to symptom improvement, and progress in the line cancellation test.

A 69-year-old man experienced cerebral infarction 8 years before initiating MNT®, with recurrence at 3 and 4 years after the first stroke. Figure 1 shows the brain computed tomography (CT) image of the patient. Brain CT conducted after his third stroke showed no significant cerebral atrophy (Figure 1A). Figures 1B,C show cerebral infarction in the right occipital lobe and left internal capsule, respectively. This case presents a complex neurological picture resulting from multiple lesions. The right hemiplegia was attributed to a lesion in the left internal capsule, affecting the pyramidal tract. The left hemispatial neglect was attributed to a lesion in the right cerebral cortex. Occlusion of the distal right middle cerebral artery may result in hemispatial neglect without contralateral paresis, as observed in our patient's right hemisphere lesion. Approximately 1 year before MNT® initiation, the patient moved to a geriatric residential care facility affiliated with our hospital. He displayed moderate right hemiplegia and severe left hemispatial neglect. His primary means of mobility within the facility was a wheelchair, which he operated independently. He began receiving physical therapy to improve walking and occupational therapy to address postural transformation and balance related to his post-stroke condition but not specifically for hemispatial neglect. Both the physical and occupational therapists noted the patient's significant neglect symptoms but were unable to address them during their allocated therapy sessions due to scheduling constraints. The case timeline (Figure 2) outlines the patient's history, step-by-step approach implemented in response to symptom improvement, and progress made in the line cancellation test.

## 3 Assessment and intervention

The first author, a board-certified music therapist, joined the hospital approximately 1 year after the patient's admission. At the request of the patient's occupational therapist, weekly individual MNT® sessions were initiated to address his severe left hemispatial neglect. At the time, his score on the line cancellation test (17) was 33. On this test, the patients are required to cross out 40 lines that are randomly orientated on an A4 sheet of paper. The number of uncrossed lines out of 40 was recorded to assess the patient's progress, with a reduced score indicating an improvement in hemispatial neglect. His Mini-Mental State Examination (MMSE) (18) score was 17, suggesting a decline in cognitive function, and dementia was suspected. Regarding ADLs, egocentric neglect was observed by both care and rehabilitation staff, especially during eating and wheelchair operations.

**Figure 3:**
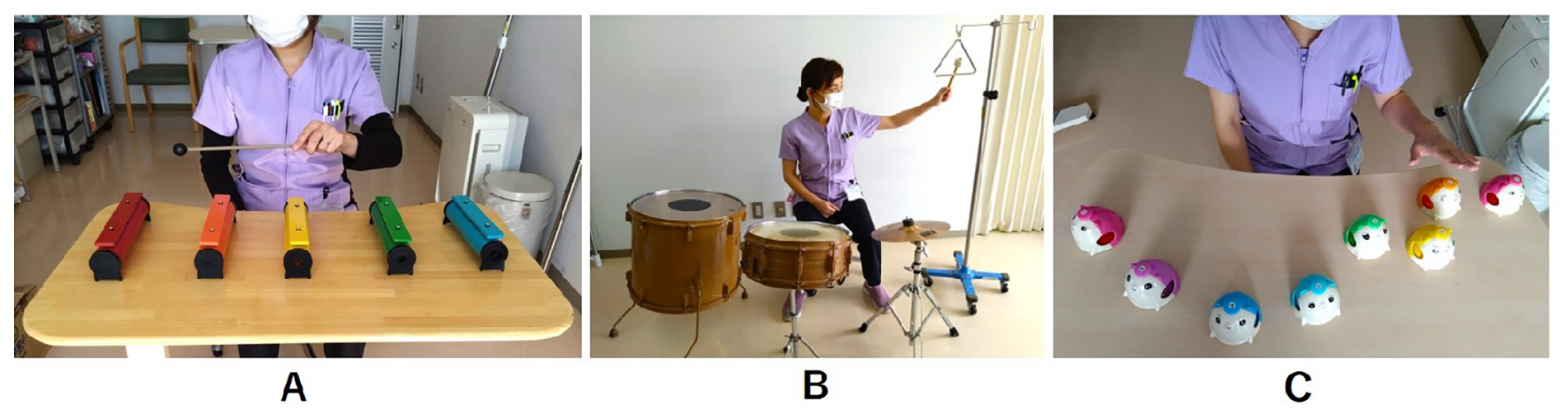
In the first few sessions, the therapist initially placed all the musical instruments (tone bars) in the patient's right recognizable space. **(A)** The therapist gradually moved the instruments to his left side, crossing his midline in a stepwise manner. **(B)** A floor tom drum, small drum, cymbal, and triangle hanging from a drip pole (as an example) were placed in a semicircle in front of the patient. **(C)** The desk bell exercise with a few bells arranged in the lower left space.

Interventions for hemispatial neglect were implemented weekly for 18 months from July 2019 to January 2021, followed by 6 months of evaluations. Tonal and percussion instruments were used, for which each tone could be independently spatially rearranged and played one at a time (Figure 3). While the patient presented with both right hemiplegia and left hemispatial neglect, the intervention specifically targeted the neglect symptoms using MNT®. The MNT® was adapted to enable the patient to participate using his unaffected left hand, focusing on exercises designed to enhance attention to the neglected side. The therapist used an electronic piano or small instruments, such as tambourines or hand drums, to provide auditory cues. The music comprised improvisations or familiar tunes with chords matching the note sequence played by the patient. As he had no musical background, simple instruments and predictable note/chord sequences were used to avoid additional cognitive load, focusing solely on directing attention to the neglected side. At the end of each session, a singing activity was performed at the patient's request, as karaoke was one of his hobbies before the stroke. This served as a reward and motivator for rehabilitation. He selected a song from a list of favorites, with the lyrics displayed on his left side.

For the initial assessment and progress evaluation, we used the line cancellation test, widely employed to assess hemispatial neglect, and the MMSE, which is regularly administered to patients at the facility. These tests were conducted 1 month after the intervention started, every 3 months during the intervention phase, and 1, 3, and 6 months after the intervention was terminated during the follow-up phase because our facility's policy required a progress report every 3 months.

At the beginning of the intervention, the patient struggled to recognize and explore musical instruments within his left hemispace. To facilitate his engagement, the therapist initially positioned all instruments within his recognizable space. For instance, the tone bars (do, re, mi, fa, so) were arranged sequentially on the right side of the table, and the patient was instructed to play them using a mallet with his unaffected hand. The therapist provided music featuring harmonic progressions alongside melodic cues, guiding him to play the notes from right to left.

Once the patient became familiar with the tone sequence, the therapist gradually shifted the bars to the left, crossing his midline (Figure 3A). Clear musical cues were utilized to encourage the patient to visually explore the left side and recognize any missed notes. He was able to self-correct when the therapist paused the music, indicating he had overlooked the leftmost tone bar. When he played without looking at bars, verbal prompts were provided to encourage him to direct his gaze toward the bars.

**Figure 4:**
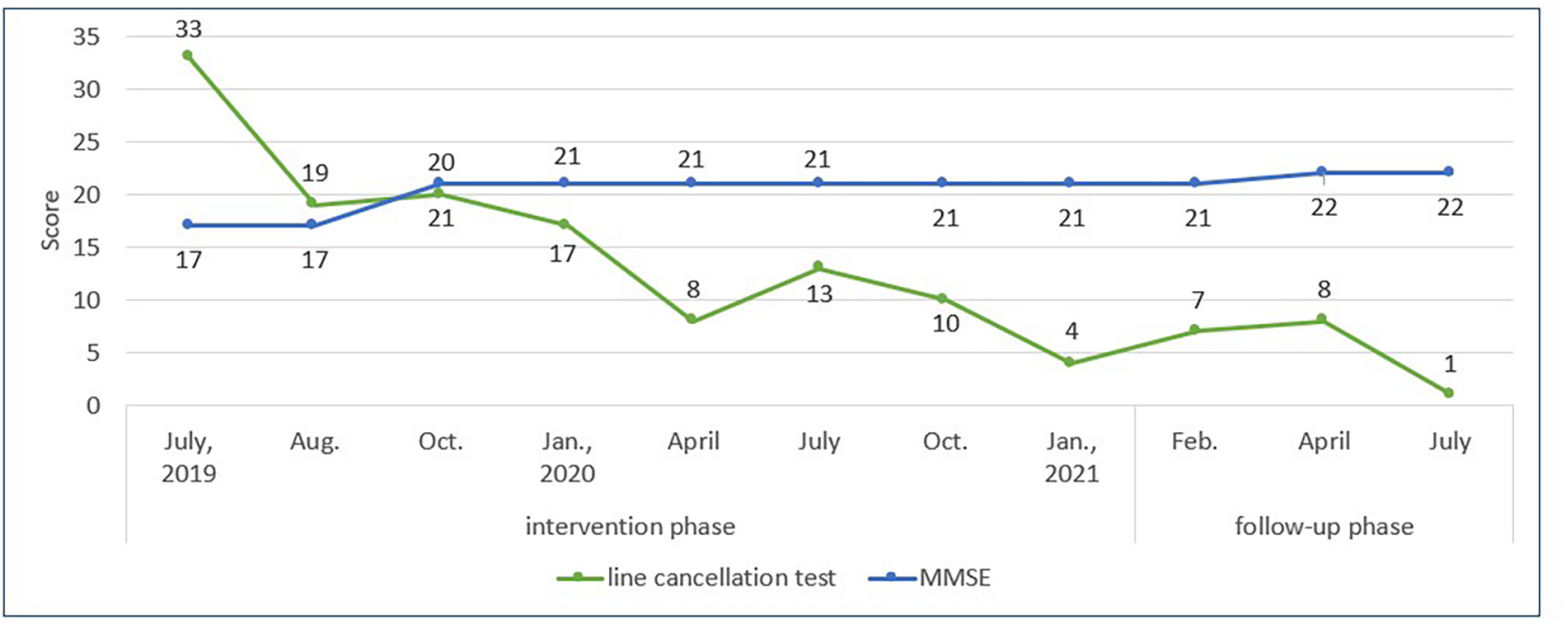
Progress over the intervention and follow-up phases. The green line shows the patient's progress in the line cancellation test, demonstrating improvement in left hemispatial neglect. The blue line represents the Mini-Mental State Examination (MMSE) results over time, which shows stable cognitive function.

Over 1 month, the frequency of missing instruments placed in the patient's left space gradually decreased. Improvement was observed in the line cancellation test (score = 19, Figure 4). Accordingly, the number of tone bars placed on the desk was increased from five to eight (an octave), encouraging him to direct his attention to a wider space on the left. At 3 months, the therapist introduced an exercise with a floor tom drum and other instruments placed in a semicircle in front of the patient (Figure 3B) and a left–right alternating strike exercise with two paddle drums held and moved by the therapist. These exercises were expected to gradually expand the patient's attention, not only on flat surfaces but also in larger spaces.

At 9 months, the patient showed improvement in the line cancellation test (score = 8) and ADLs. He was less likely to forget to brake on the left side of the wheelchair and no longer approached the left wall when maneuvering to the session room. However, verbal prompts from the care staff were needed when self-feeding because he often missed the plates on the tray's lower left side. This phenomenon was also observed in the line cancellation test; he did not cross out the lines in the test sheet's lower left section.

Thus, at 10 months, the therapist introduced the desk bell exercise. The bells are smaller than tone bars, allowing for the patient's attention to be directed to a more specific and restricted area when appropriately arranged on the desk. The therapist increased the difficulty of the visual search by changing the arrangement from a horizontal row to shifting each bell backward or forward and placing a few bells in the lower left space (Figure 3C). The bells were arranged to play a simple, repetitive counter line to a song familiar to the patient and were tapped to play in sequence from the patient's right to the left side. Furthermore, in the alternating strike exercise, the frequency with which the paddle drums were presented in the lower left space around the patient increased. Initially, his responses to stimuli in the lower left space were slow or missed more often than those in other areas. However, over the course of approximately 3 months, these delays and missed responses gradually decreased. By continuing the intervention and adjusting the cognitive load to maintain the patient's motivation, the number of missed responses in the lower left area showed improvement at the 18-month evaluation (score = 4). In contrast, the scores declined at 12 and 15 months (score = 13 and 10, respectively).

## 4 Follow-up and outcomes

The intervention was terminated after 18 months to investigate whether the effects would last. Follow-up evaluations at 1, 3, and 6 months with scores of 7, 8, and 1, respectively, showed no decline in progress (Figure 4). Regarding ADLs, the patient's difficulty in paying attention to the plates on the left side of the food tray improved. This was partly attributed to the collaborative work of the care staff, who routinely encouraged the patient to touch and count all the plates with his unaffected hand when served a meal.

In the present case, an 18-month individual MNT® in the chronic phase of severe left hemispatial neglect resulted in sustained symptom improvement, measured using the line cancellation test and observed ADL performance. The patient demonstrated excellent compliance and commitment to the intervention throughout the intervention period without missing any scheduled sessions for personal reasons or refusing to attend. However, some sessions were canceled during the summer vacation period and over the New Year's holiday due to therapist unavailability. These cancellations were due to the scheduled vacations of the therapists and were not related to the willingness or ability of the patient to participate. Figure 4 shows the patient's progress on the line cancellation test and MMSE over the intervention and follow-up phases.

## 5 Discussion

This case study demonstrated that active instrument playing with music stimulation benefited a patient with left hemispatial neglect. The left-side neglect greatly improved, even in the chronic phase, indicated by a decrease of the uncrossed lines in the line cancellation test. Improvements were observed in specific ADLs, particularly in wheelchair management (operation and brake manipulation) and self-feeding tasks, demonstrating enhanced awareness and function on the neglected left side. Our results align with those of previous reports supporting the effectiveness of music intervention with active instrument playing (11–13). Further, a recent systematic review of music-based interventions for hemispatial neglect found only short-term effects (6). The present report demonstrates that improvements persisted for 6 months after therapy termination. While the temporary decline in scores observed at 12 and 15 months post-therapy initiation may indicate a training-induced ceiling effect, suggesting that the patient had reached a peak in neglect reduction during that period, the subsequent improvements and sustained benefits observed at the 6-month follow-up indicate that the intervention had lasting effects extending beyond those anticipated from a ceiling effect. During the follow-up phase after the final session, the patient did not receive any rehabilitation training specifically for hemispatial neglect. However, routine physical and occupational therapy sessions continued as part of the patient's standard care. Although these sessions were not directly targeted at addressing neglect symptoms, they may have contributed to the patient's overall recovery. This continuation of standard rehabilitation practices should be considered when interpreting the follow-up results, particularly considering the observed ceiling effect. In the present case, after a temporary plateau in scores, the residual left lower spatial neglect improved after a gradual improvement in left hemispatial neglect, although many patients with improved left hemispatial neglect have residual left lower spatial neglect (3).

In interventions for hemispatial neglect, guiding the patient to spontaneously direct his or her attention to the neglected side is essential (3). Active instrument playing with musical cues encourages spontaneous attention switching from right to left and vice versa through appropriate instrument placement in the space. Nevertheless, therapists must clearly consider each case individually and devise ways to adapt to each patient's progress during treatment. In this case, to promote spontaneous attention control and self-awareness of neglect, the therapist required careful observation of the patient's responses and real-time decision-making regarding the timing of musical cues, insertion of brief pauses, and repositioning of instruments to expand the spatial field. These real-time adjustments were crucial in guiding the patient's attention effectively. The strategic use of musical cues and pauses helped create moments of anticipation and reflection (19), potentially enhancing the patient's engagement with the neglected side. By dynamically altering the spatial configuration of instruments, the therapist aimed to gradually expand the patient's attention field, tailoring the therapy to align with the patient's progress. Moreover, structured patterns in the time, rhythm, and sequence of the music played by the therapist likely enhanced the patient's spontaneous responses and actions by providing cognitive–perceptual feedback. This enhancement was likely achieved by integrating various instruments and predictable patterns of auditory stimuli, guiding spatial attention and orientation to the neglected side and encouraging intentional movements (10). These factors may have contributed to the improvement of the patient's attention to the entire neglected area and in addressing partial neglect.

Although hemispatial neglect is a symptom that is considered difficult to generalize to ADLs (3), we observed an improvement in egocentric neglect during the patient's ADLs. The impact of the intervention on the line cancellation test, which greatly differs from musical tasks, may indicate some recovery of the attentional network in the brain. Another report has documented the generalization to ADLs (tooth brushing) as a result of music therapy with active instrument playing (14). The use of active instrument playing may be a potential approach for improving hemispatial neglect by providing multisensory stimulation and facilitating neuroplasticity (15, 16, 20). In this chronic case, MNT® with active instrument playing reduced persistent perceptual attention deficits, and follow-up assessments suggested that these effects persist for at least 6 months.

Another factor that may have contributed to the favorable outcomes in this case is that the patient had right-side paralysis, implying that the unaffected left side was the side of hemispatial neglect. Considering the influence of sensory input, it is possible that attention to the left direction during the intervention, where his left hand could move and receive tactile feedback from the instrument, was more easily achieved. A few studies have reported that moving patients' affected left upper limb in their left space and having them observe the movements improves left hemispatial neglect (21, 22).

Our study has some limitations. We used only the line cancellation test from the Behavioural Inattention Test (BIT) to minimize patient burden, although the BIT includes various hemispatial neglect assessments. As individuals with hemispatial neglect often exhibit significant performance variability (3, 7, 12), utilizing all tests in BIT could enhance symptom understanding. In this case, more tests might have yielded deeper insights into the patient's symptoms, potentially leading to better interventions. Future studies should incorporate multiple assessments, such as clock drawing and line cutting tests, to achieve a more comprehensive evaluation of hemispatial neglect. Using multiple assessments on a statistically significant number of participants may help identify which types are more likely to benefit from MNT® and the differing durations of intervention required. Additionally, we did not employ the ADL scale to assess changes in daily functioning, despite observations from care staff, rehabilitation staff, and the music therapist indicating improvements in the patient's ability to operate a wheelchair and eat independently. Future studies should assess the frequency of behaviors caused by hemispatial neglect during ADLs to better understand changes. The Catherine Bergego Scale is suitable for assessing patients with hemispatial neglect, as it incorporates both self-assessment and evaluator observations of disabilities in daily lives (23). This may help tailor intervention plans to the patient's distress. Finally, the findings of this study, which focused on a patient with right hemiplegia and left hemispatial neglect, may not apply to those with left hemiplegia and/or right hemispatial neglect. Further research is needed to explore how intervention effects vary by lesion site, side and severity of neglect and paralysis, time since onset, type of music intervention (active or passive), and impact on ADLs in multiple participants.

In conclusion, the present case shows that even chronic post-stroke severe hemispatial neglect symptoms can be improved with the use of auditory cues and the appropriate placement of musical instruments during active instrument playing.

## 6 Patient perspective

The patient's wife provided the following account: “I am very happy that my husband was able to receive music therapy at the facility and that one of the aftereffects of his stroke has improved. When I visit him, he looks brighter than before, and I believe it is because of the music therapy”.

## Data Availability

The original contributions presented in the study are included in the article, further inquiries can be directed to the corresponding author.
